# Parahippocampal Cortex Mediates the Relationship between Lutein and Crystallized Intelligence in Healthy, Older Adults

**DOI:** 10.3389/fnagi.2016.00297

**Published:** 2016-12-06

**Authors:** Marta K. Zamroziewicz, Erick J. Paul, Chris E. Zwilling, Elizabeth J. Johnson, Matthew J. Kuchan, Neal J. Cohen, Aron K. Barbey

**Affiliations:** ^1^Decision Neuroscience Laboratory, University of Illinois Urbana-ChampaignUrbana, IL, USA; ^2^Beckman Institute for Advanced Science and Technology, University of Illinois Urbana-ChampaignUrbana, IL, USA; ^3^Neuroscience Program, University of Illinois Urbana-ChampaignUrbana, IL, USA; ^4^Jean Mayer USDA Human Nutrition Center on Aging, Tufts UniversityBoston, MA USA; ^5^Research, Scientific and Medical Affairs, Abbott NutritionColumbus, OH, USA; ^6^Department of Psychology, University of Illinois Urbana-ChampaignUrbana, IL, USA; ^7^Carle Neuroscience Institute, Carle Foundation HospitalUrbana, IL, USA; ^8^Department of Internal Medicine, University of Illinois Urbana-ChampaignUrbana, IL, USA; ^9^Department of Speech and Hearing Science, University of Illinois Urbana-ChampaignUrbana, IL, USA; ^10^Institute for Genomic Biology, University of Illinois Urbana-ChampaignChampaign, IL, USA

**Keywords:** lutein, parahippocampal cortex, crystallized intelligence, cognitive aging, nutritional cognitive neuroscience

## Abstract

**Introduction:** Although, diet has a substantial influence on the aging brain, the relationship between dietary nutrients and aspects of brain health remains unclear. This study examines the neural mechanisms that mediate the relationship between a carotenoid important for brain health across the lifespan, lutein, and crystallized intelligence in cognitively intact older adults. We hypothesized that higher serum levels of lutein are associated with better performance on a task of crystallized intelligence, and that this relationship is mediated by gray matter structure of regions within the temporal cortex. This investigation aims to contribute to a growing line of evidence, which suggests that particular nutrients may slow or prevent aspects of cognitive decline by targeting specific features of brain aging.

**Methods:** We examined 76 cognitively intact adults between the ages of 65 and 75 to investigate the relationship between serum lutein, tests of crystallized intelligence (measured by the Wechsler Abbreviated Scale of Intelligence), and gray matter volume of regions within the temporal cortex. A three-step mediation analysis was implemented using multivariate linear regressions to control for age, sex, education, income, depression status, and body mass index.

**Results:** The mediation analysis revealed that gray matter thickness of one region within the temporal cortex, the right parahippocampal cortex (Brodmann's Area 34), partially mediates the relationship between serum lutein and crystallized intelligence.

**Conclusion:** These results suggest that the parahippocampal cortex acts as a mediator of the relationship between serum lutein and crystallized intelligence in cognitively intact older adults. Prior findings substantiate the individual relationships reported within the mediation, specifically the links between (i) serum lutein and temporal cortex structure, (ii) serum lutein and crystallized intelligence, and (iii) parahippocampal cortex structure and crystallized intelligence. This report demonstrates a novel structural mediation between lutein status and crystallized intelligence, and therefore provides further evidence that specific nutrients may slow or prevent features of cognitive decline by hindering particular aspects of brain aging. Future work should examine the potential mechanisms underlying this mediation, including the antioxidant, anti-inflammatory, and membrane modulating properties of lutein.

## Introduction

As the older adult population expands, the economic burden of care and treatment of age-related health disorders also rises. Between 2015 and 2060, the United States will experience significant growth of its older population, with the size of the population aged 65 and over more than doubling from an estimated 46 million in 2015 to 98 million in 2060 (Mather et al., 2015). Therefore, successful strategies to promote healthy brain aging are of significant interest to public health initiatives in the United States.

Nutrition is a promising target for intervention efforts to support healthy brain aging (Zamroziewicz and Barbey, 2016). Accumulating evidence indicates that particular nutrients may slow or prevent aspects of age-related cognitive decline by targeting specific features of brain aging. Studies that couple neuroimaging techniques with neuropsychological testing provide insight into mechanisms of action through which particular nutrients might influence specific aspects of age-related cognitive decline (Bowman et al., 2012; Zamroziewicz et al., 2015; Boespflug et al., 2016; Gu et al., 2016). While some nutrients may be effective at preventing late-life changes in the brain, other nutritional factors may accumulate across the lifespan and therefore confer neuroprotection in the aging brain (Söderberg et al., 1990; Coyle and Puttfarcken, 1993). Identifying the mechanisms through which nutrients provide neuroprotective effects will help guide the development of successful lifelong dietary strategies for healthy brain aging.

Carotenoids are naturally occurring pigments made by plants, and can only be acquired through the diet (Erdman et al., 2015). Xanthophylls are a subclass of carotenoids, which have a polar molecular structure and therefore possess unique membrane-spanning properties (Erdman et al., 2015; Widomska et al., 2016). As compared to the other dietary carotenoids, xanthophylls preferentially accumulate in neural tissue, with lutein accounting for the majority of carotenoid accumulation in the brain (Craft et al., 2004; Johnson, 2012; Johnson et al., 2013; Li et al., 2014; Vishwanathan et al., 2014b; Widomska et al., 2016). As the most prevalent carotenoid in the brain, lutein is thought provide a variety of neuroprotective benefits. Candidate mechanisms of action are primarily based on the unique membrane spanning properties of lutein and include influencing membrane properties, such as fluidity, interneuronal communication via gap junctions, ion exchange, oxygen diffusion, membrane stability, and preventing oxidation and inflammation (Stahl and Sies, 1996, 2001; Paiva and Russell, 1999; Krinsky, 2002; Izumi-Nagai et al., 2007; Johnson, 2014; Widomska and Subczynski, 2014; Erdman et al., 2015). Lutein accretes in the brain across the entire lifespan, and may therefore contribute to brain health in a lifelong manner (Renzi et al., 2014; Bovier and Hammond, 2015; Lieblein-Boff et al., 2015). Notably, lutein is selectively distributed in gray matter, and has been detected in the prefrontal cortex, the temporal cortex, and the hippocampus (Craft et al., 2004; Vishwanathan et al., 2014b). Blood levels of lutein correlate with brain concentrations of lutein in older adults, suggesting that blood concentrations can serve a measure of lutein status in the brain (Johnson et al., 2013).

Lutein status has been linked to cognitive performance across the lifespan (Feeney et al., 2013; Johnson et al., 2013; Renzi et al., 2014; Vishwanathan et al., 2014a; Bovier and Hammond, 2015). Of particular interest, lutein levels have been linked to memory and general intelligence (Feeney et al., 2013; Johnson et al., 2013; Vishwanathan et al., 2014a), which are cognitive constructs closely related to crystallized intelligence. Crystallized intelligence refers to the ability to retrieve and use information that has been acquired throughout life (Horn and Cattell, 1966). Although most aspects of cognitive function undergo age-related decline, certain aspects of cognition—like crystallized intelligence—are spared and even show improvement with age (Craik and Bialystok, 2006; Park and Reuter-Lorenz, 2009). Measuring crystallized intelligence may therefore be a way to assess the lifelong impact of nutritional factors, rather than the immediate effects of nutrients on preventing age-related decline (Craik and Bialystok, 2006).

Crystallized intelligence is dependent upon the temporal cortex, and particular regions of the temporal cortex may play key roles in implementing this cognitive function (Colom et al., 2009; Barbey et al., 2012). In general, structural integrity of the temporal cortex is associated with performance on tasks of crystallized intelligence, but integrity of specific regions within the temporal cortex may underlie the preservation of this cognitive function in aging (Choi et al., 2008). For example, the parahippocampal cortex plays a role in mediating the storage of knowledge about objects (Ricci et al., 1999; Aminoff et al., 2013). This region of the temporal cortex shows resistance to age-related structural decline in the absence of neurodegenerative disease, unlike other regions within the temporal cortex that show significant cortical thinning even in healthy aging (Salat et al., 2004; Jiang et al., 2014). Although the sparing of particular regions within the temporal cortex may be linked to the preservation of crystallized intelligence in healthy aging, the question remains: do particular neuroprotective nutrients like lutein underlie this sparing of cognition and brain health?

In summary, increasing evidence suggests that lutein may be a reliable nutrient biomarker for healthy brain aging: (i) among the carotenoids, lutein accounts for the majority of accumulation in the brain and provides unique neuroprotective benefits, (ii) lutein status has been linked to cognitive performance across the lifespan, and (iii) lutein is selectively distributed in gray matter of brain regions known to underlie the preservation of cognitive function in healthy brain aging. However, the core brain structures upon which lutein may act to preserve cognition have not been investigated. Prior research suggests that lutein plays a neuroprotective role across the lifespan, crystallized intelligence is resistant to age-related decline, and specific regions of the temporal cortex may underlie the preservation of crystallized intelligence. This study aims to explore the role of structures within the temporal cortex in mediating the relationship between serum lutein levels and crystallized intelligence in healthy older adults.

## Materials and methods

### Participants

This cross-sectional study enrolled 122 participants (ages 65–75) from Carle Foundation Hospital, a local and readily available cohort of well-characterized older adults. No participants were cognitively impaired, as defined by a score of lower than 26 on the Mini-Mental State Examination (Folstein et al., 1975). Participants with a diagnosis of mild cognitive impairment, dementia, psychiatric illness within the last 3 years, stroke within the past 12 months, and cancer within the last 3 years were excluded. Participants were also excluded for current chemotherapy or radiation, an inability to complete study activities, prior involvement in cognitive training or dietary intervention studies, and contraindications for magnetic resonance imaging (MRI). Of these 122 participants, 76 subjects had a complete dataset at the time of data analysis, including neuropsychological testing, MRI, and serum analysis.

### Standard protocol approval and participant consent

This study was approved by the University of Illinois Institutional Review Board and, in accordance with the stated guidelines, all participants read and signed informed consent documents.

### Serum acquisition and lutein analysis

Serum was prepared for extraction using 100 μL of sample and 0.5 mL 0.9% saline. Echinenone, in ethanol, was added as an internal standard (DSM Nutritional Products). The mixture was extracted by using 2 mL CHCL_3_:CH_3_OH (2:1, vol/vol). The mixture was vortexed and then centrifuged at 800 g for 15 min at 4°C. The CHCl_3_ layer was removed and evaporated to dryness under nitrogen. A second extraction was performed on the mixture using 3 mL hexane. The mixture was vortexed and centrifuged as above. The hexane layer was combined with the first extraction and evaporated to dryness under nitrogen. The residue was redissolved in 100 μL of ethanol, vortexed, and sonicated for 30 s. A 20 μL aliquot was used for HPLC analysis.

Lutein was measured using a reversed-phase, gradient HPLC system. The system consisted of a Waters Alliance 2695 Separation Module LC pump, auto-sampler, Waters 2996 Photo-Array Detector (Millipore, Milford, MA) and a semi-bore C30 column (3 μm, 150 × 4.6 mm, YMC, Wilmington, NC). The chromatographic separations were performed on a Waters Alliance 2695 (Millipore, Milford, MA) system using a UV detector and Waters Empower Pro software. The flow rate was 0.4 mL/min and the gradient elution used two mixtures of methanol, tert-butyl methyl ether, and water (mixture A: 83/15/2; v/v/v), mixture B: 8/90/2, v/v/v). The gradient procedure was: 0 to 1 min 100% A, 1 to 8 min linear gradient to 70% A, 8 to 13 min 70% A, 13 to 22 min linear gradient to 45% A, 22 to 24 min 45% A, 24 to 34 min linear gradient to 5% A, 34 to 38 min 5% A, 38 to 40 min linear gradient to 100% A, and 40 to 50 min 100% A.

The method yielded adequate separation of lutein, which was then quantified at 445 nm. Quantification was determined from peak areas in the HPLC chromatograms calibrated against known amounts of standards. Concentrations were corrected for extraction and handling losses by monitoring the recovery of the echinenone internal standard. The lower detection with this method is 0.2 pmol. A composite lutein score was computed by summing across all-*trans* lutein and *cis*-lutein.

### Neuropsychological tests

Crystallized intelligence was measured by the Wechsler Abbreviated Scale of Intelligence–second edition (WASI-II) (Wechsler, 1999). This assessment measured crystallized intelligence by way of a verbal comprehension index, which was the product of two subtests: a vocabulary subtest and a similarities subtest. In the vocabulary subtest, participants were asked to verbally define vocabulary words (i.e., What does lamp mean?) that became progressively more challenging. In the similarities subtest, participants were asked to relate pairs of concepts (i.e., How are a cow and bear alike?) that became progressively more challenging. These subtests were compiled into a verbal comprehension index, which provided a measure of acquired knowledge, verbal reasoning, and attention to verbal information, and therefore served as a marker of crystallized intelligence.

### Volumetric brain MRI

Volumetric analysis was performed on data from a 3D high-resolution (0.9 mm isotropic) T1-weighted scan using MPRAGE acquisition. Cortical reconstruction was performed with the Freesurfer image analysis suite, which is documented and freely available for download online (http://surfer.nmr.mgh.harvard.edu/). The technical details of these procedures are described in prior publications (Dale and Sereno, 1993; Dale et al., 1999; Fischl et al., 1999a,b, 2001, 2002, 2004; Fischl and Dale, 2000; Fischl, 2004; Ségonne et al., 2004; Han et al., 2006; Jovicich et al., 2006; Reuter et al., 2010, 2012). All cortical reconstructions were manually checked for accuracy, as recommended by the software developers. The volumetric analyses focused on gray matter volume in the temporal cortex provided by Freesurfer parcellation. Regions of interest included the superior temporal cortex, middle temporal cortex, inferior temporal cortex, banks of the superior temporal sulcus, fusiform cortex, transverse temporal cortex, entorhinal cortex, temporal pole, and parahippocampal cortex.

### Covariates

Covariates were included according to previous association with cognitive decline. These included age (continuous), gender (nominal, man/woman), education (nominal, five fixed levels), income (nominal, six fixed levels), body mass index (continuous, hereafter BMI), and depression status (nominal, yes/no). Although all participants had received a diagnosis of no depression at enrollment, the SF-36 Health Survey (Ware et al., 1993) revealed four participants with symptoms consistent with depression, and in accordance with other studies, this was considered in the analysis as a covariate. Temporal cortex gray matter volume (continuous) was also included as a covariate in mediation analyses to assess the relationship between specific regions within the temporal cortex, serum lutein, and crystallized intelligence. These covariates were included in each of the three steps of the mediation analysis.

### Statistical analyses

**Figure 1:**
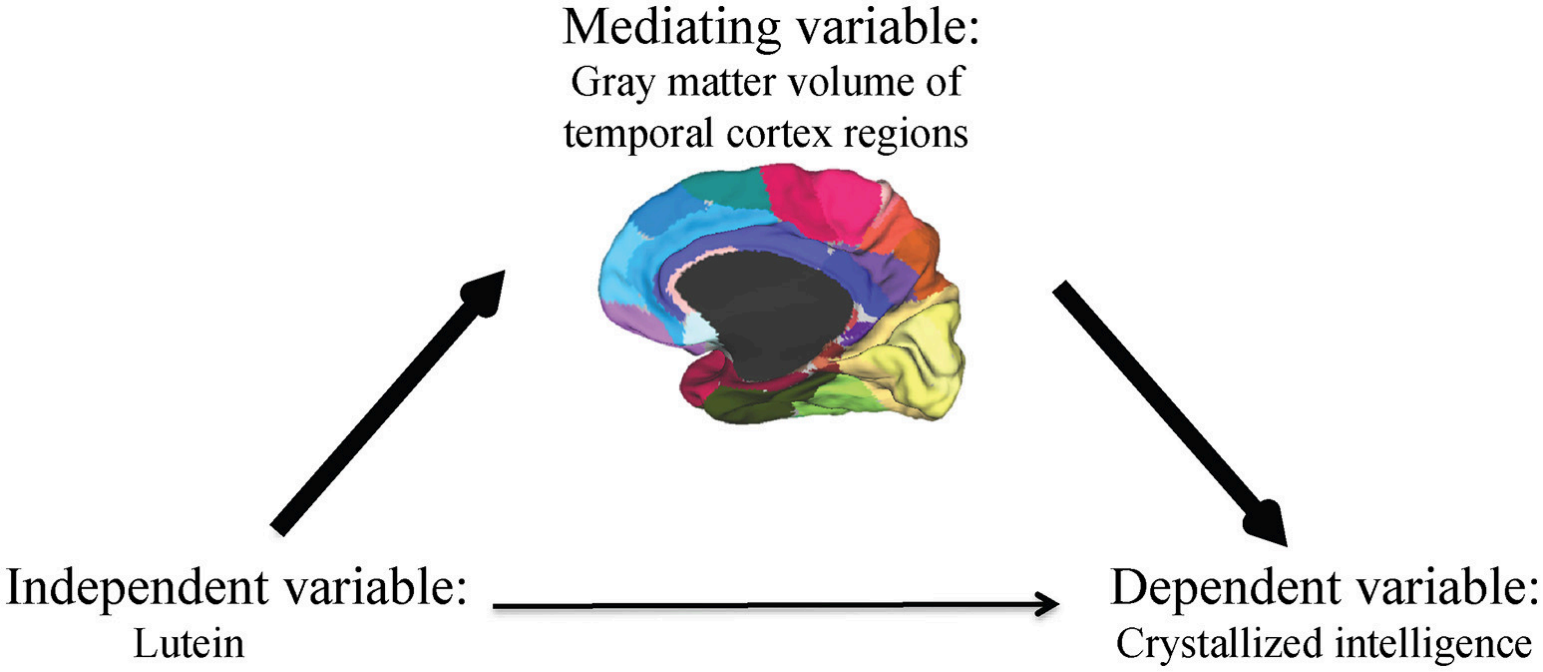
**The primary requirement for mediation is a significant indirect mediation effect, defined as the effect of the independent variable (lutein) through the mediator (gray matter volume of regions within the temporal cortex) on the dependent variable (crystallized intelligence)**.

A formal mediation analysis was used in an effort to better understand the relationship between serum lutein, gray matter volume of regions within the temporal cortex, and crystallized intelligence using a three-step framework. The primary requirement for mediation is a significant indirect mediation effect, Zhao et al. (2010) defined as the effect of the independent variable (lutein) through the mediator (gray matter volume of regions within the temporal cortex) on the dependent variable (crystallized intelligence) (Figure 1).

Statistics were performed in SPSS Statistical Packages version 23 (SPSS, Inc., Chicago, IL, USA), and mediation analyses were performed using the *indirect* macro designed for SPSS (Preacher and Hayes, 2008). In the first step, a regression model was used to characterize the relationship between serum lutein and gray matter volume of regions within the temporal cortex (path a), controlling for the covariates listed above and applying a false discovery rate (FDR) correction for multiple comparisons (*q* < 0.05, one-tailed). In the second step, a regression model was used to characterize the relationship between serum lutein and crystallized intelligence (path c), controlling for the covariates listed above. In the third step, the *indirect* macro was used to implement the bootstrapping method to estimate mediation effects. This analysis drew 1000 bootstrapped samples with replacement from the dataset to estimate a sampling distribution for the indirect and direct mediation effects, controlling for the covariates listed above. The indirect mediation effect refers to the pathway from serum lutein to gray matter volume of temporal cortex regions to crystallized intelligence (path a to b). The direct mediation effect refers to the direct pathway from serum lutein to crystallized intelligence (path c'). A statistically significant full mediation that matches the hypothesized framework is indicated by: (i) an indirect mediation effect that does not include zero within 95% bias-corrected confidence intervals, and (ii) a direct mediation effect that does include zero within 95% bias-corrected confidence intervals. A statistically significant partial mediation, (i.e., the mediation does not account for all possible mediators) is indicated by: (i) an indirect mediation effect that does not include zero within 95% bias-corrected confidence intervals, and (ii) a direct mediation effect that does not include zero within 95% bias-corrected confidence intervals (Zhao et al., 2010). Results are reported using unstandardized regression coefficients (β) and statistical significance (*p*) for each individual regression relationship, and a 95% bias-corrected confidence interval (95% CI) for the direct and indirect effects of the mediation.

## Results

### Participant characteristics

Participants had a mean age of 69 years and 67 percent of participants were females. The mean lutein level was 454 pmol/mL. The mean MMSE score was 29 and the Wechsler Abbreviated Scale of Intelligence crystallized intelligence score was 111 (Table 1).

**Table 1:** **Characteristics of study population**.

**Demographics**	**Total *n* = 76**
Age, years (mean ± std; median; range)	69 ± 3; 69; 65–75
Female, n (%)	50(67%)
Education, n (%)	1(1) some high school
	11(15) high school degree
	12(16) some college
	52(68) college degree
Income, n (%)	1(1) < $15,000
	2(3) $15,000–$25,000
	13(17) $25,000–$50,000
	17(22) $50,000–$75,000
	18(24) $75,000–$100,000
	25(33) > $100,000
Depression, n (%)	72(95) no
	4(5) yes
**Serum nutrients**	**(pmol/mL − mean ± std; median; range)**
Lutein	454 ± 275; 418; 120–1328
**Psychometrics**	**(mean ± std)**
Mini-Mental State Examination	29 ± 1
Crystallized intelligence score	111 ± 14
**Volumetric MRI (gray matter volume)**	**(mm − mean ± std)**
Left temporal lobe	6670 ± 645
Right temporal lobe	5458 ± 599
Left superior temporal	10889 ± 1482
Right superior temporal	10858 ± 1502
Left middle temporal	9593 ± 1418
Right middle temporal	10724 ± 1445
Left inferior temporal	10105 ± 1606
Right inferior temporal	9665 ± 1401
Left banks of the superior temporal sulcus	2229 ± 464
Right banks of the superior temporal sulcus	2179 ± 409
Left fusiform	9281 ± 1445
Right fusiform	8973 ± 1397
Left transverse temporal	1102 ± 210
Right transverse temporal	836 ± 164
Left entorhinal	1779 ± 388
Right entorhinal	1729 ± 369
Left temporal pole	2464 ± 383
Right temporal pole	2247 ± 342
Left parahippocampal	2067 ± 318
Right parahippocampal	1914 ± 258

### Serum lutein and crystallized intelligence, mediated by parahippocampal structure

The mediation analyses indicated that out of all regions within the temporal cortex, gray matter thickness of only the right parahippocampal cortex (Brodmann's Area 34) mediated the relationship between serum lutein and crystallized intelligence. Each relationship within the mediation is described below in a stepwise fashion.

**Figure 2:**
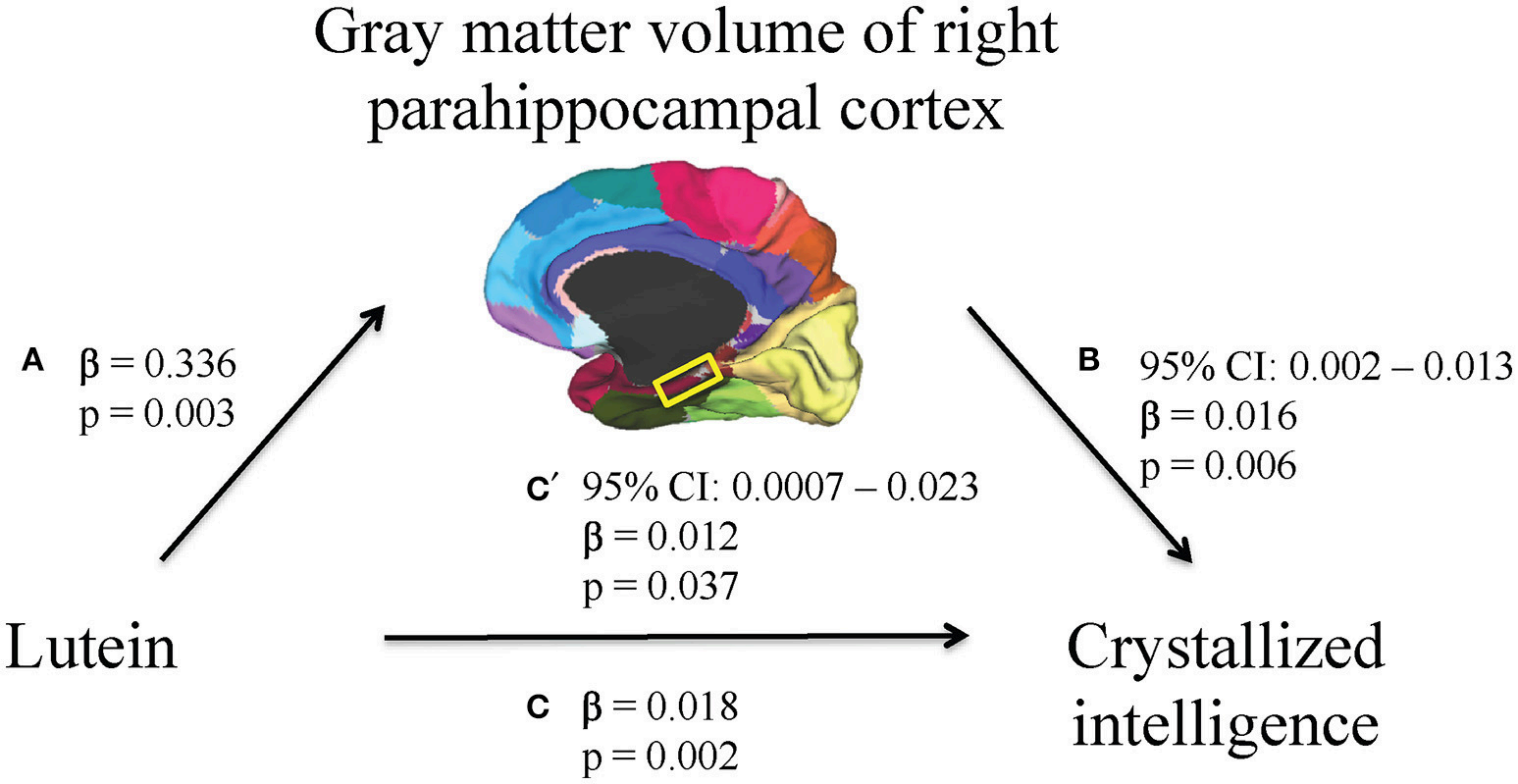
**A mediation model was used to characterize the relationship between serum lutein, gray matter volume of regions within the temporal cortex, and crystallized intelligence**. Serum lutein concentrations positively associated with gray matter volume of the right parahippocampal cortex (path a). Serum lutein positively associated with crystallized intelligence (path c). The indirect pathway of mediation (i.e., the effect of serum lutein through gray matter volume of the right parahippocampal cortex on crystallized intelligence; path a to b) was statistically significant. Likewise, the direct pathway of mediation (i.e., the effect of serum lutein on crystallized intelligence; path c') was statistically significant. Therefore, gray matter volume of the right parahippocampal cortex partially mediated the relationship between serum lutein and crystallized intelligence.

First, higher serum lutein levels were associated with larger volume of one region within the temporal cortex, the right parahippocampal cortex (β = 0.336, *p* = 0.003, Table 2). Therefore, the relationship between serum lutein concentration and gray matter volume of the right parahippocampal cortex was considered in the context of the mediation model (β = 0.336, *p* = 0.003, Figure 2, path a). Second, higher serum levels of lutein associated with superior crystallized intelligence (β = 0.018, *p* = 0.002, Figure 2, path c). Third, mediation effects were estimated for the right parahippocampal cortex. The indirect pathway of mediation was significant (95% CI: 0.002–0.013, β = 0.016, *p* = 0.006, Figure 2, path a to b), and the direct pathway of mediation was significant (95% CI: 0.0007–0.023, β = 0.012, *p* = 0.037, Figure 1, path c'). Therefore, the right parahippocampal cortex partially mediated the relationship between serum lutein and crystallized intelligence (Figure 2).

**Table 2:** **Multivariate linear regressions show relationship between serum lutein levels and volume of regions within the temporal cortex, controlling for age, gender, education, income, body mass index, depression status, and total temporal cortex volume**.

**Cortical region volume**	**β**	***p***
Left superior temporal	−0.424	0.318
Right superior temporal	−0.378	0.364
Left middle temporal	−0.809	0.084
Right middle temporal	0.421	0.356
Left inferior temporal	0.922	0.093
Right inferior temporal	0.186	0.695
Left banks of the superior temporal sulcus	0.013	0.943
Right banks of the superior temporal sulcus	−0.051	0.713
Left fusiform	−0.318	0.442
Right fusiform	−0.328	0.432
Left transverse temporal	−0.086	0.393
Right transverse temporal	−0.171	0.03
Left entorhinal	0.331	0.037
Right entorhinal	−0.148	0.151
Left temporal pole	0.265	0.095
Right temporal pole	0.133	0.457
Left parahippocampal	0.106	0.443
Right parahippocampal	0.336	0.003^*^

*Results are reported using unstandardized regression coefficients (β) and statistical significance (p) for each individual regression relationship*.

* *p-values that survive FDR correction for multiple comparisons (p < 0.05, one-tailed)*.

## Discussion

This study revealed that gray matter volume of the right parahippocampal cortex mediates the relationship between serum lutein and crystallized intelligence. This report provides a novel link between lutein, gray matter volume of a specific cortical region, and crystallized intelligence. The individual relationships reported within the mediation, including those between serum lutein levels and parahippocampal cortex (Figure 2, path a), between serum lutein levels and crystallized intelligence (Figure 2, path c), and between parahippocampal cortex and crystallized intelligence (Figure 2, path b), are each supported by prior findings reviewed in turn below.

Prior findings support each relationship reported within the mediation model. The first relationship demonstrated a positive association between serum lutein and gray matter volume of the parahippocampal cortex in the right hemisphere (Figure 2, path a). Four lines of evidence support this finding: (i) serum lutein correlates with brain concentrations of lutein in older adults, (ii) lutein preferentially accumulates in brain tissue, (iii) xanthophylls are selectively distributed in gray matter, as opposed to white matter, and (iv) lutein is found in the temporal cortex (Craft et al., 2004; Johnson, 2012; Johnson et al., 2013; Vishwanathan et al., 2014b). Second, higher serum levels of lutein were associated with higher scores of crystallized intelligence (Figure 2, path c). Prior work demonstrates that intake of green leafy and cruciferous vegetables—both major dietary sources of lutein—is positively associated with cognitive function (Kang et al., 2005; Morris et al., 2006). Indeed, macular pigment optical density (MPOD), a biomarker of lutein concentrations in the brain, has been reported to be significantly related to cognitive function in three independent research groups (Feeney et al., 2013; Vishwanathan et al., 2013, 2014a, 2016; Renzi et al., 2014). Among the carotenoids, lutein most consistently associates with measures of intelligence and memory (Johnson et al., 2008, 2013). Third, the indirect pathway of mediation indicated a mediatory effect of right parahippocampal gray matter volume on the relationship between lutein and crystallized intelligence (Figure 2, path a to b). Previous studies indicate that greater temporal cortex gray matter volume correlates with general intelligence, and the parahippocampal cortex in particular shows activity during tasks that require auditory-verbal recall (Grasby et al., 1993; Colom et al., 2009). Furthermore, healthy aging is characterized by the preservation of crystallized intelligence as well as structural integrity of the parahippocampal cortex (Horn and Cattell, 1966; Salat et al., 2004; Jiang et al., 2014). The unilateral nature of this mediation is supported by work suggesting that the right hemisphere is more resistant to age-related and disease-related neurodegeneration than the left hemisphere (Chételat et al., 2005; Querbes et al., 2009; Risacher et al., 2010; Mosconi et al., 2014).

The underlying physiological mechanisms of the relationship between serum lutein levels, crystallized intelligence, and cortical integrity of the parahippocampal cortex are 3-fold. First, lutein is differentially localized to membrane domains high in polyunsaturated fatty acids, and is therefore well positioned to prevent oxidation of vulnerable lipids (Widomska and Subczynski, 2014). Second, by preventing lipid oxidation, lutein may help preserve long-chain polyunsaturated fats, such as docosahexaenoic acid (DHA), for cleavage and conversion into anti-inflammatory compounds (Miller et al., 2014). Third, lutein is a polar and soluble molecule, and can therefore span the membrane and influence membrane properties, such as fluidity, ion exchange, oxygen diffusion, membrane stability, and interneuronal communication via gap junctions (Stahl and Sies, 2001). Importantly, these neuroprotective effects are relevant across the lifespan, and may therefore be particularly important for the preservation of cognitive functions that are enhanced with increasing age, such as crystallized intelligence (Horn and Cattell, 1966; Bovier and Hammond, 2015; Lieblein-Boff et al., 2015).

The partial nature of this mediation is supported by the specificity of this analysis, which focused on one aspect of cognition and its underlying cortical structures. Given that lutein is thought to influence a wide range of biological processes in the brain, it is unlikely that the role of lutein in the aging brain is limited to crystallized intelligence and the parahippocampal cortex (Erdman et al., 2015). Rather than claiming the parahippocampal cortex as the sole mediator of the relationship between lutein and crystallized intelligence, this study suggests that preserving structural integrity of the parahippocampal cortex is one mechanism through which lutein contributes to the preservation of cognitive function. These findings add to a growing line of evidence which suggests that particular nutrients may slow or prevent aspects of age-related cognitive decline by targeting specific features of brain aging (Bowman et al., 2012; Zamroziewicz et al., 2015; Gu et al., 2016). In the case of lutein, future studies are needed to assess whether lutein is uniquely protective of crystallized intelligence and temporal cortex structure, or whether other carotenoids contribute neuroprotective effects as well. Another promising direction for future work is to examine the interactive effects among nutrients through the use of nutrient biomarker pattern analysis—a technique that enables an investigation of the beneficial effects of broader nutrient profiles on healthy brain aging. Ultimately, this line of research can inform a comprehensive and personalized approach to nutritional intervention that takes into account dietary patterns and individual variability in nutritional status and brain health.

The strengths of the present study include: (i) the use of blood biomarkers to measure physiological status of lutein, (ii) the use of structural magnetic resonance imaging to measure regional cortical integrity with high spatial resolution, and (iii) the assessment of a particular component of cognitive function known to be highly variable across individuals, rather than a global cognitive function measure with little variability within a sample. The limitations of this study include: (i) relatively small sample size, (ii) cross-sectional design, (iii) lack of MPOD measurement to better reflect lutein concentrations in neural tissue (Vishwanathan et al., 2013, 2016), and (iv) lack of assessment of other carotenoids. Thus, directions for future research include: (i) larger samples to confirm these results, (ii) longitudinal studies to investigate the relationship between serum lutein and crystallized intelligence in different stages of life, (iii) MPOD measures of lutein in neural tissue, and (iv) assessment of other carotenoids to determine potential neuroprotective benefits.

## Author contributions

MZ is the primary author on this manuscript. AB is the primary investigator and contributed to study design and manuscript drafting. EP, CZ, EJ, MK, and NC contributed to study design and manuscript drafting.

### Conflict of interest statement

MK is an employee of Abbott Nutrition. The other authors declare that the research was conducted in the absence of any commercial or financial relationships that could be construed as a potential conflict of interest.
